# Targeted Capture of Hundreds of Nuclear Genes Unravels Phylogenetic Relationships of the Diverse Neotropical Palm Tribe Geonomateae

**DOI:** 10.3389/fpls.2019.00864

**Published:** 2019-07-12

**Authors:** Oriane Loiseau, Ingrid Olivares, Margot Paris, Marylaure de La Harpe, Anna Weigand, Darina Koubínová, Jonathan Rolland, Christine D. Bacon, Henrik Balslev, Finn Borchsenius, Angela Cano, Thomas L. P. Couvreur, César Delnatte, Frédérique Fardin, Marc Gayot, Fabian Mejía, Talita Mota-Machado, Mathieu Perret, Julissa Roncal, Maria José Sanin, Fred Stauffer, Christian Lexer, Michael Kessler, Nicolas Salamin

**Affiliations:** ^1^Department of Computational Biology, University of Lausanne, Lausanne, Switzerland; ^2^Department for Systematic and Evolutionary Botany, University of Zurich, Zurich, Switzerland; ^3^Centre for Biodiversity and Environment Research, University College London, London, United Kingdom; ^4^Department of Biology, Unit Ecology and Evolution, University of Fribourg, Fribourg, Switzerland; ^5^Department of Botany and Biodiversity Research, University of Vienna, Vienna, Austria; ^6^Natural History Museum of Geneva, Geneva, Switzerland; ^7^Department of Zoology, University of British Columbia, Vancouver, BC, Canada; ^8^Department of Biological and Environmental Sciences, University of Gothenburg, Gothenburg, Sweden; ^9^Gothenburg Global Biodiversity Centre, Gothenburg, Sweden; ^10^Department of Bioscience, Biodiversity and Ecoinformatics, Aarhus University, Aarhus, Denmark; ^11^Science Museums, Aarhus University, Aarhus, Denmark; ^12^Cambridge University Botanic Garden, Cambridge, United Kingdom; ^13^IRD, DIADE, University of Montpellier, Montpellier, France; ^14^National Forestry Office, Fort-de-France, France; ^15^Parc National de la Guadeloupe, Guadeloupe, France; ^16^National Forestry Office, Guadeloupe, France; ^17^Facultad de Ciencias y Biotecnología, Universidad CES, Medellin, Colombia; ^18^Programa de Pós-Graduação em Biologia Vegetal, Departamento de Botânica, Instituto de Ciências Biológicas, Universidade Federal de Minas Gerais, Belo Horizonte, Brazil; ^19^Department of Botany and Plant Biology, Conservatory and Botanical Garden of the City of Geneva, University of Geneva, Geneva, Switzerland; ^20^Department of Biology, Memorial University of Newfoundland, St. John’s, NL, Canada

**Keywords:** Arecaceae, *Geonoma*, Neotropics, phylogenetic informativeness, phylogenomics, species complexes

## Abstract

The tribe Geonomateae is a widely distributed group of 103 species of Neotropical palms which contains six ecologically important understory or subcanopy genera. Although it has been the focus of many studies, our understanding of the evolutionary history of this group, and in particular of the taxonomically complex genus *Geonoma*, is far from complete due to a lack of molecular data. Specifically, the previous Sanger sequencing-based studies used a few informative characters and partial sampling. To overcome these limitations, we used a recently developed Arecaceae-specific target capture bait set to undertake a phylogenomic analysis of the tribe Geonomateae. We sequenced 3,988 genomic regions for 85% of the species of the tribe, including 84% of the species of the largest genus, *Geonoma*. Phylogenetic relationships were inferred using both concatenation and coalescent methods. Overall, our phylogenetic tree is highly supported and congruent with taxonomic delimitations although several morphological taxa were revealed to be non-monophyletic. It is the first time that such a large genomic dataset is provided for an entire tribe within the Arecaceae. Our study lays the groundwork not only for detailed macro- and micro-evolutionary studies within the group, but also sets a workflow for understanding other species complexes across the tree of life.

## Introduction

Palms (Arecaceae) are an important ecological component (Henderson, 2002; Couvreur et al., 2011) and a useful plant group of tropical ecosystems (Macia et al., 2011; Gruca et al., 2015). The palm family was recently advocated as a model group to understand the evolution of tropical rain forests (Baker and Couvreur, 2013) and numerous studies have investigated their phylogenetic relationships and systematics (Uhl and Dransfield, 1987; Baker et al., 1999; Dransfield et al., 2008; Baker and Dransfield, 2016). However, given the remarkably low rate of molecular evolution observed in palms (Wilson et al., 1990), phylogenetic studies at different taxonomic levels within the Arecaceae based on a few plastid or nuclear genes generally result in poorly resolved phylogenetic trees, especially at the species level (Roncal et al., 2005, 2008; Cuenca et al., 2008; Baker et al., 2011; Bacon et al., 2012a, 2016a,b, 2017; Meerow et al., 2015; Sanín et al., 2016).

The lack of informative genetic markers, combined with insufficient taxonomic sampling, currently limits our understanding of the phylogenetic relationships within the most diverse palm genera in the Neotropics, such as *Bactris* Jacq. ex Scop.*, Chamaedorea* Willd., and *Geonoma* Willd. These three genera are mostly small shade-adapted palms and they contain the most abundant palm species in the understory of many Neotropical forests (Vormisto et al., 2004; Balslev et al., 2016, 2017; Ley-lopez and Avalos, 2017; Muscarella et al., 2018). They also often exhibit a high amount of intraspecific phenotypic variation (Roncal, 2006), which is hard to address with a taxonomic classification. This is exemplified by *Geonoma*, which, with 68 recognized species (Henderson, 2011), is the third most diverse palm genus in the Neotropics. *Geonoma* belongs to the tribe Geonomateae Luerss., together with five other genera. These five additional genera range in size from two (*Welfia* H. Wendl.) to 21 species (*Calyptrogyne* H. Wendl.), and are also small understory palms except for *Calyptronoma* Griseb. (three species) and *Welfia*, which can reach up to 15 m and 25 m, respectively. The tribe displays a wide geographical and ecological distribution, occurring from southern Mexico to south-eastern Brazil, including the Caribbean, with species growing from the lowlands up to 3,000 m elevation in the Andes. The tribe has been intensively studied and its main biological aspects, such as taxonomy (Wessels Boer, 1968; Zona, 1995; Stauffer et al., 2003; Henderson, 2005, 2011, 2012; Henderson and Villalba, 2013), ecology (Chazdon, 1992; Knudsen et al., 1998; Sampaio and Scariot, 2008; Pizo and Almeida-Neto, 2009), and phylogenetic relationships (Roncal et al., 2005, 2010, 2011, 2012) have been characterized to some extent. Considerable research has also been dedicated to investigate the phenotypically widely variable species complexes that represent 20% of the species of *Geonoma* (Borchsenius, 2002; Henderson and Martins, 2002; Roncal, 2006; Roncal et al., 2007; Henderson, 2011; Borchsenius et al., 2016). Despite all these efforts, the evolutionary history of *Geonoma* and Geonomateae remains only partially understood due to the paucity of DNA sequences, which so far are available only for three nuclear loci and approximately 60% of the species in the tribe.

Obtaining a robust phylogenetic hypothesis for the Geonomateae is therefore crucial to enable a reliable assessment of the systematic relationships of its lineages, but also to provide the foundation to assess the macroevolutionary patterns and the dynamics of diversification in this key palm group. The increasing affordability of next generation sequencing techniques, which offers the possibility to sequence hundreds of loci at a time, has already benefitted many plant phylogenetic studies (e.g., Nicholls et al., 2015; Sass et al., 2016; Mandel et al., 2017; Mitchell et al., 2017). For Arecaceae, while most of genome-scale data initially focused on commercially important species such as the oil palm (Uthaipaisanwong et al., 2012; Singh et al., 2013) and the date palm (Yang et al., 2010; Al-Mssallem et al., 2013), evolutionary biologists have put considerable effort in the last few years to generate genomic data across the whole family and are aiming at a species level phylogenetic tree of all palms (Comer et al., 2015, 2016; Heyduk et al., 2015; Barrett et al., 2016, 2018).

In this context, the recent development of several sequence capture kits for the Arecaceae (Heyduk et al., 2015; de La Harpe et al., 2019) represents an ideal opportunity to fill the gaps in palm phylogenomics. Here, using the bait kit developed by de La Harpe et al. (2019), we sequenced 4,184 genomic regions for 85% of the species of tribe Geonomateae, including 84% of the species of *Geonoma* and applied both standard and coalescent-based methods to reconstruct the phylogenetic relationships within the tribe. Using substantial intraspecific sampling, we assessed the validity of the species delimitations proposed by Henderson (2011) for the widespread and highly morphologically variable species complexes. We also estimated the phylogenetic informativeness of the DNA regions in the capture kit and proposed a smaller selection of the most useful genomic regions for phylogenetic studies at deep and shallow evolutionary scales within the Arecaceae. Our results show that these new molecular tools increase our understanding of the systematics and evolution in this important group of understory palms and open up new directions of research to test hypotheses about the factors underlying the diversification of species in palms.

## Materials and Methods

### Taxon Sampling

We gathered a total of 312 samples of either silica-dried leaves or herbarium fragments from specimens stored at the herbarium of Geneva (G) and the Herbario Nacional Colombiano (COL), including 240 samples representing 57 (84%) of the 68 currently recognized species of *Geonoma* (Supplementary Table S1). Among the 11 missing species of *Geonoma*, eight are narrow endemics known only from the type collections (*G. deneversii* A. J. Hend., *G. dindoensis* A. J. Hend., *G. gentryi* A. J. Hend. and *G. operculata* A. J. Hend.) or less than ten herbarium specimens (*G. peruviana* A. J. Hend., *G. sanmartinensis* A. J. Hend., *G. schizocarpa* A. J. Hend. and *G. venosa* A. J. Hend.). Whenever possible, we sampled several individuals per species and included different subspecies. For widely distributed species, sample selection was designed to cover the greatest possible extant of their geographic distribution. Our sampling also included 65 individuals representing 25 species from the other five genera of the tribe Geonomateae (100% taxon sampling for *Asterogyne* H. Wendl, 61% for *Calyptrogyne*, 100% for *Calyptronoma*, 75% for *Pholidostachys* H. Wendl. Ex Hook. f., and 50% for *Welfia*), covering in total 85% of the tribe’s species richness. For the purpose of computing the phylogenetic informativeness of the targeted genomic regions across the whole Arecaceae, we also included seven samples from phylogenetically more distant palm genera, belonging to subfamilies Arecoideae Burnett (*Bactris, Cocos* L., *Socratea* H. Karst, and *Wettinia* Poepp.), Ceroxyloideae Drude (*Ceroxylon* Bonpl. ex DC.), and Coryphoideae Burnett (*Licuala* Wurmb).

### DNA Extraction, Dual-Indexed Library Preparation, and Target Capture Sequencing

DNA was extracted using the DNeasy^®^ plant mini kit (Qiagen, Venlo, Netherlands) following the supplier’s instructions. DNA quality and degradation were evaluated with agarose gels and a Nanodrop^TM^ TM spectrophotometer ND-1000 (Thermo Fisher Scientific, Waltham, MA, United States) and DNA was quantified with a Qubit^®^ Fluorometer v 2.2 (Thermo Fisher Scientific, Waltham, MA, United StatesUSA). When possible, a total of 500 ng of DNA were used per sample for library preparation.

DNA samples were fragmented to 400 bp fragments with a bioruptor^®^ ultrasonicator UCD-200TM-EX (Diagenode, Liège, Belgium) with six cycles of 30 s ON, and 90 s OFF. This step was omitted for samples with degraded DNA. Library preparations were performed following de La Harpe et al. (2019). Briefly, sample cleaning, end repair and A-tailing steps were carried out with a KAPA LTP library preparation kit (Roche, Basel, Switzerland), and adaptor ligation and adaptor fill-in reactions steps (Meyer and Kircher, 2010).

A set of 60 dual-index primers were used for amplification, as recommended by Kircher et al. (2011), to avoid inaccuracies in multiplex sequencing. Two sets of 7 bp indexes were generated using the create_index_sequences.py Python program (Meyer and Kircher, 2010): one set of 30 indexes for the P5 Illumina primers, and one set of 30 indexes for the P7 Illumina primers. The index lists were chosen to contain a balanced subset of indexes with an edit distance of 4 to reduce the chance of conversion by sequencing and amplification errors. Adaptor and primer sequences are described in Supplementary Table S2. Eight cycles of PCR were used for most samples, except for 29 low quality and degraded samples for which 12 cycles of PCR were necessary to obtain sufficient DNA amount (Supplementary Table S1). Libraries were quantified with a Qubit^®^ Fluorometer v 2.2. Target capture was performed using the custom kit PopcornPalm developed by de La Harpe et al. (2019) and deposited in Dryad^1^. This kit targets 4,051 genes and 133 non-genic putatively neutral regions. Target capture was conducted on pooled dual-indexed libraries following myBait^®^ Custom Target Capture Kits protocol v3.0 (Arbor Biosciences, Ann Arbor, MI, United States), with 18 h incubation time at 65°C and 12 cycles of post-capture PCR reactions. Pools of 64 samples were used as template for each target capture hybridization reaction, using an initial amount of 1.2 μg of pooled libraries. The pooled target capture reactions were quantified with a Qubit^®^ Fluorometer v 2.2 before sequencing with an Illumina HiSeq3000 sequencer in paired-end 2 × 150 bp mode.

### Read Trimming, Mapping, and SNP Calling

Reads were first trimmed with the program condetri v2.2 (Smeds and Künstner, 2011) using a base quality score of 20 as high-quality threshold parameter before mapping to the *Geonoma undata* Klotzsch pseudoreference genome described in de La Harpe et al. (2019) with bowtie2 v2.2.5 (Langmead and Salzberg, 2012) and the very-sensitive-local option. Only reads that mapped at a unique location in the genome were kept for analysis.

Before variant calling, PCR duplicates were masked with the software Picard v1.119^2^, and reads were realigned around indels and base-recalibrated using GATK v3.8 (McKenna et al., 2010). SNPs were then called for targeted genomic regions using UnifiedGenotyper of GATK v3.8 using the EMIT_ALL_SITES option in order to obtain the full sequence of the targets. The main advantage of paired-end 2 × 150 bp read sequencing is the potential recovery of adjacent regions to the exonic targets. For this reason, the entire sequence including UTRs, exons and introns was called for each gene. Sites were filtered with the following parameters using VCFtools v0.1.13 (Danecek et al., 2011): minimum quality >20, no indel allowed, minimum depth of 8× per sample, and maximum of 50% of missing data. For each genomic region the alignment in fasta format was generated using the program vcf-tab-to-fasta^3^.

### Selection of Most Informative Genomic Regions

**FIGURE 1:**
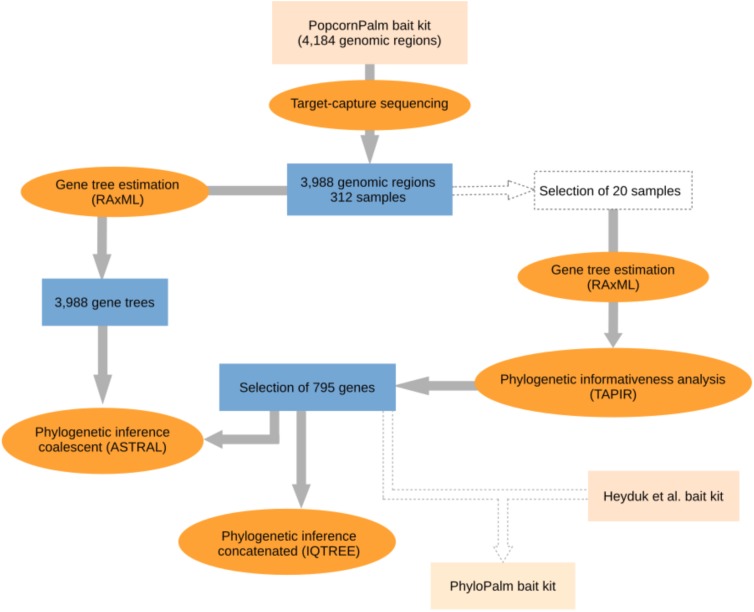
Flowchart illustrating the steps of the phylogenomic analyses. The selection of 795 genes was combined with the bait kit from Heyduk et al. (2015) to provide a new bait kit for future phylogenomic studies.

Because the bait kit developed by de La Harpe et al. (2019) for micro- and macro-evolutionary analyses in palms is large (over 4,000 genomic regions) and contains several fast-evolving DNA regions that are not necessarily useful for phylogenomic studies, we selected a subsample of the most informative genomic regions which we then used to infer the species tree of the Geonomateae. Additionally, we made available a new bait kit for future phylogenomic studies in palms, which combines the subset of genes presented here with the genes from the Heyduk et al.’s kit (2015). Our workflow for gene selection and phylogenomic analyses is summarized in Figure 1. In order to maximize the phylogenetic informativeness of the retained genes for the Arecaceae and not only for tribe Geonomateae, the selection steps were performed on a dataset which contained species from three different Arecaceae subfamilies (Arecoideae, Ceroxyloideae, and Coryphoideae). First, we estimated the phylogenetic informativeness for each gene at different geological time intervals with the program TAPIR (Pond and Muse, 2005; Townsend, 2007; Faircloth et al., 2012). For each alignment, TAPIR estimates the site rates under the best-fitting substitution model and further computes a quantitative measure of the power of the gene to resolve the branching order at different depths of a given phylogenetic tree. To reduce computing time, the analysis was performed on a subset of 20 out of the 312 samples sequenced, which were selected to represent a wide range of evolutionary time scales, from intra-specific variability up to 88 Ma of divergence. The selection included three species of *Geonoma* (including four samples of *G. deversa*), two species of *Asterogyne*, two species of *Calyptrogyne*, as well as *Welfia regia* H. Wendl., *Bactris gasipaes* Kunth, *Cocos nucifera* L., *Socratea exorrhiza* (Mart.) H. Wendl, *Wettinia maynensis* Spruce, *Ceroxylon alpinum* Bonpl. ex DC., and two species of *Licuala*. Because TAPIR does not accept missing data, we only considered the genes for which sequence data were available for all 20 samples. Details for this analysis can be found in the Supplementary Material. Then, we selected the most appropriate genes for phylogenomic analyses according to the following criteria: (1) single-copy genes, (2) genes located on one of the 16 chromosomes of the *Elaeis guineensis* Jacq. reference genome (i.e., no gene on the extra low quality scaffolds), (3) genes absent from the bait kit of Heyduk et al. (2015) to avoid redundancy in the final bait set, (4) genes among the top 500 genes with the highest phylogenetic informativeness measure and/or with the highest mean bootstrap value per gene tree, (5) genes with a minimum mean bootstrap value per gene tree >60, and (6) genes with a minimum of five baits covering their exonic regions. We constrained our selection to a total of 17,091 baits to obtain a maximum of 20,000 baits when combined with the 2,909 baits of the Heyduk’s kit (Heyduk et al., 2015). This option thus allows for coherence among different studies and maximizes the informativeness of the data at the lowest possible cost, since the smallest kit size available at the Arbor Biosciences company (Ann Arbor, MI, United States) is of 20,000 baits.

### Phylogenetic Inference

Phylogenetic trees were estimated using both maximum likelihood and coalescent based methods. We used the software IQ-TREE (Nguyen et al., 2015) to estimate, under the maximum likelihood criteria, the topology and branch lengths of the phylogenetic tree for all samples based on the concatenated analysis of the reduced set of genes satisfying the criteria described above. We partitioned the data by gene (Chernomor et al., 2016), using a GTR+GAMMA model of substitutions for each gene, and estimated support using the ultrafast bootstrap option (Hoang et al., 2018). We did not perform model testing because parameter rich models such as GTR+G and GTR+G+I have been shown in simulations to suffice for phylogeny reconstruction (Hoff et al., 2016; Abadi et al., 2019). The consensus tree obtained from this analysis was visualized using Figtree v1.4.3 (Rambaut, 2012). Next, we applied a coalescent approach which takes into account gene tree incongruence due to incomplete lineage sorting (Liu et al., 2009). We used ASTRAL 4.10.12, a two-step coalescent-based method that estimates the species tree given a set of gene trees (Mirarab et al., 2014; Mirarab and Warnow, 2015). All gene trees were first estimated with RaxML 8.2.10 (Stamatakis, 2014), using the GTR+GAMMA model of substitution and support estimated with the -autoMRE option. We then performed ASTRAL on the reduced data set and obtained a measure of branch support by computing local posterior probabilities. The impact of including weakly informative genes in two-step coalescent analyses is debated and while some studies showed that it can help to resolve difficult nodes (Blom et al., 2017) others argued that it reduces the accuracy of species tree estimation (Liu et al., 2015). To test whether including less informative genes would improve our phylogenetic inference we also applied ASTRAL to our full genomic dataset. Additionally, we computed quartet support values to measure the level of gene tree incongruence in our dataset and plotted quartet support for the three possible topologies at each branch using a python script^4^. All trees were rooted using the two *Licuala* species as outgroup.

## Results

### Target Capture Sequencing

In total, we recovered DNA sequences for 3,988 genomic regions out of 4,184. On average, we obtained 2,064,810 reads per sample (Supplementary Table S1). After filtering, a total of 7,438,988 high quality bases including 2,288,308 SNPs were obtained with an average coverage of 30.8× and only 9.3% of missing data. When considering only the samples of *Geonoma*, 1,102,445 SNPs were recovered.

### Phylogenetic Informativeness

**FIGURE 2:**
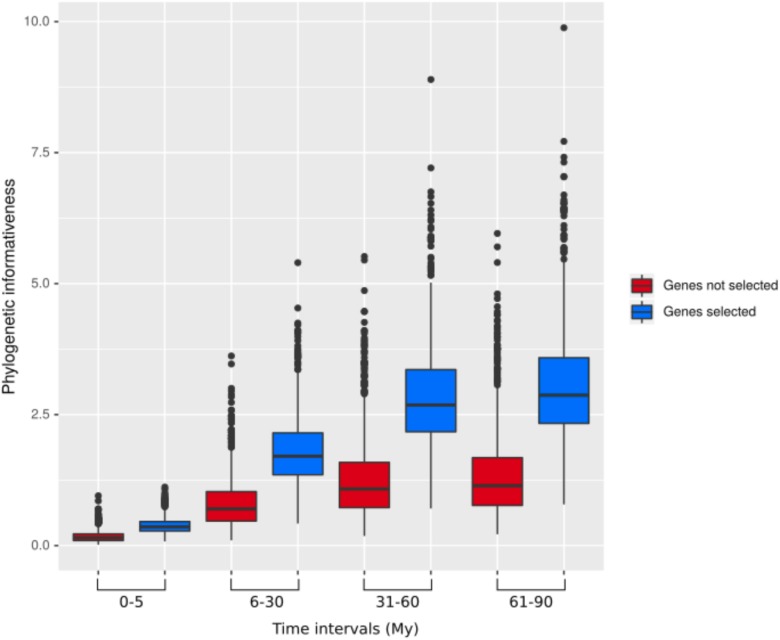
Phylogenetic informativeness of the 795 selected genes (blue) and the remaining genes (red) over different evolutionary time intervals.

Across our data set, phylogenetic informativeness increased with increasing evolutionary divergence times (Figure 2). After applying the selection step, the reduced dataset of 17,091 baits contained 795 genes, ranging from 1,108 to 12,710 bp in length. The corresponding bait kit combining our 795 genes with Heyduk’s baits (Heyduk et al., 2015) is available at the Arbor Biosciences company (Ann Arbor, MI, United States) under the name “PhyloPalm.”

### Phylogenetic Inference

**FIGURE 3:**
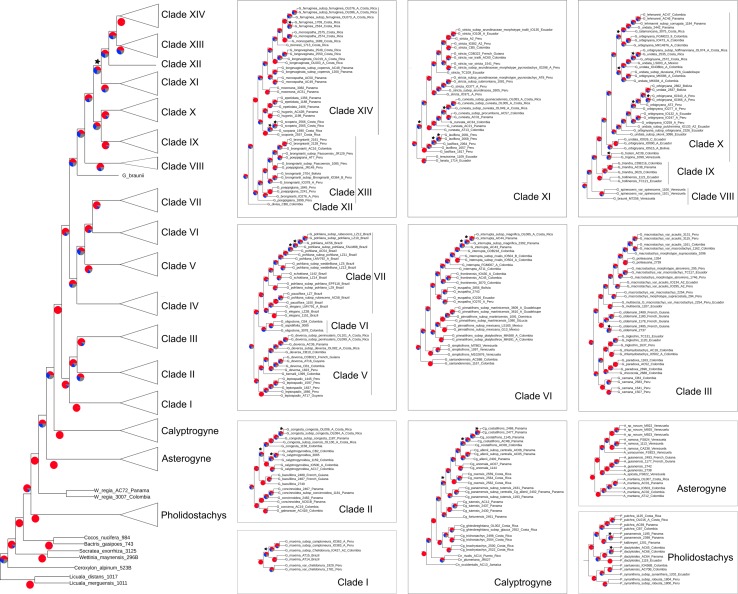
Cladogram inferred with ASTRAL on the set of 795 gene trees. Pie charts indicate for each branch the percentage of gene trees aggreing with the topology of the species tree (red) and the percentage of gene trees supporting the other two alternative topologies (blue and gray). Stars indicate branches with LPP below 0.9.

**FIGURE 4:**
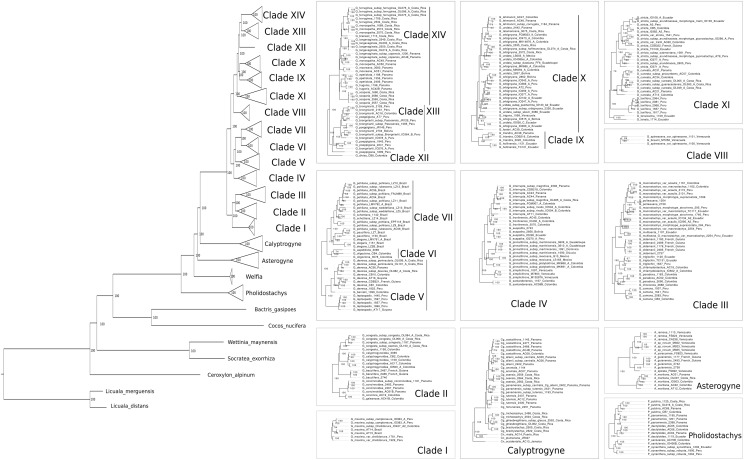
Maximum likelihood phylogeny inferred from the concatenated alignment of the 795 selected genes. Numbers indicate BS.

The total length of the concatenated alignment of the 795 selected genes was 3,064,021 bp. Phylogenetic trees obtained from the different datasets and methods had largely congruent topologies, except for the sister group of Clades XII-XIV (see section “Discussion” for clades numbers). This corresponded to Clade XI in the coalescent analysis of the 795 genes (with local posterior probability [LPP] of 0.59, Figure 3) and to Clades IX-X both in the concatenated analysis (with bootstrap support [BS] of 100%, Figure 4) and the coalescent analysis of the full dataset (with LPP of 0.66). For the 795 genes dataset, the support was slightly higher in the phylogenetic tree obtained with IQ-TREE (96% of nodes with BS >90, Figure 4) than with ASTRAL (89% of LPP >0.9, Figure 3). In the coalescent analyses, support increased with the size of the gene set, with 96% of branches having a LPP >0.9 in the phylogenetic tree obtained from the complete dataset of 3,988 genes. This is expected since the LPP are dependent on the discordance among gene trees but also the number of gene trees analyzed (Sayyari and Mirarab, 2016). Additionally, quartet support values indicated that gene tree incongruence is widespread across the phylogeny (Figure 3). In all analyses *Calyptronoma* was recovered paraphyletic, with *C. plumeriana* (Mart.) Lourteig and *C. rivalis* (O.F. Cook) L.H. Bailey more closely related to *Calyptrogyne* than to *C. occidentalis* (Sw.) H.E. Moore. The remaining five genera of tribe Geonomateae were recovered as monophyletic, with BS of 100% in the maximum likelihood phylogenetic tree and posterior probabilities of 1 in the coalescent phylogenetic trees.

## Discussion

The tribe Geonomateae is an ideal group to study plant evolutionary history in Neotropical rainforests for several reasons. First, it comprises the third largest genus of all Neotropical palms. Second, its species are distributed across all habitat types along the Andean and Central-American mountains as well as the Pacific, Caribbean and Amazonian lowlands, and in many of these areas they represent an important floristic element. Finally, *Geonoma* includes several species complexes with tremendous morphological variation which renders the taxonomic delimitation of species challenging. Because of these interesting characteristics, the systematics (Henderson et al., 1995; Henderson, 2011), ecology (Chazdon, 1991; Rodriguez-Buritica et al., 2005), and evolution (Roncal et al., 2011, 2012) of *Geonoma* have received significant attention. However, previous phylogenetic analyses relied on limited taxonomic and molecular sampling, thus preventing a detailed understanding of the phylogenetic relationships within the group. In particular, the phylogenetic trees recovered by Roncal et al. (2005, 2010, 2011, 2012) were not fully resolved and the status of species complexes had not been investigated. In this study, we addressed these shortcomings by applying a target-capture approach, using the baits developed by de La Harpe et al. (2019) to sequence nearly 4,000 genes for 57 species of *Geonoma* and 25 species of the closely related genera of tribe Geonomateae. We performed concatenation and coalescent-based phylogenetic inferences which resulted in highly similar topologies, despite substantial amount of gene tree incongruence across the phylogeny. We showed that only a fraction of our complete genomic dataset was sufficient to resolve phylogenetic relationships within the Geonomateae (Figures 3, 4).

### Implications for the Systematics of Tribe Geonomateae

Phylogenetic relationships between the six genera of Geonomateae were so far poorly understood since various studies recovered different topologies (e.g., Baker et al., 2009; Roncal et al., 2010, 2011, 2012). Here, we were able to fully resolve the intergeneric relationships within the tribe but quartet support values in ASTRAL indicate a high level of gene tree incongruence (Figure 3), which problably explains the contrasted findings of the previous studies. We hypothesize that incomplete lineage sorting caused by the rapid divergence of the genera within tribe Geonomateae, as suggested by the very short branches in the maximum likelihood phylogenetic tree (Figure 4), is responsible for the observed gene tree discordance. Additionally, we confirmed the previously hypothesized paraphyly of the Caribbean endemic genus *Calyptronoma* and consequently advocate that it should be synonymized under *Calyptrogyne*, the sister group of *Geonoma.* The unique sample of *P. synanthera* (Mart.) H.E. Moore subspecies *synanthera* did not cluster with our two samples of *P. synanthera* subspecies *robusta* (Trail) A.J. Hend., but was instead recovered as sister to *P. sanluiensis* A. J. Hend. *Pholidostachys synanthera* subspecies *synanthera* and *P. sanluiensis* are Andean taxa which co-occur in the Central Cordillera of Colombia whereas *P. synanthera* subspecies *robusta* is a lowland West-Amazonian taxa. Additional sampling would be needed to test whether this placement is the result of hybridization between *P. synanthera* subspecies *synanthera* and *P. sanluiensis* or whether subspecies *synanthera* and *robusta* are actually separate species. Finally, we were able to assess the robustness of the current taxonomy for *Geonoma*, the largest and taxonomically most challenging genus of the tribe, in which the large degree of phenotypic variation has complicated species delimitations for a long time.

### Phylogenetic Clades Within *Geonoma*

**FIGURE 5:**
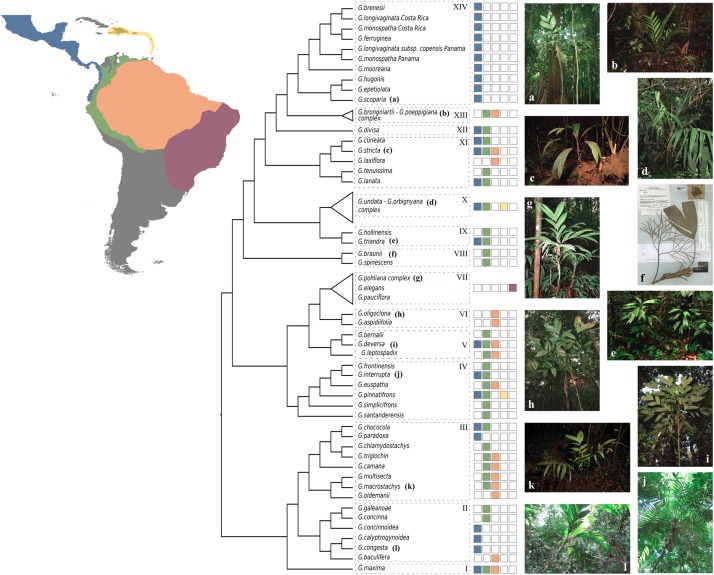
Summary cladogram of the phylogenetic relationships in *Geonoma*. Dashed boxes with numbers refer to clades mentioned in the discussion. Colored squares show the geographic distribution of species. Photos by Ingrid Olivares **(a)**, FP7-PALMS project archive **(b,c,h,i,k)**, Rodrigo Cámara Leret **(e)**, Fred Stauffer **(f)**, Oriane Loiseau **(d,j,l)**, and Talita Mota Machado **(g)**.

Based on our coalescent phylogeny and following the most recent phylogenetic reconstructions of the genus (Henderson, 2011; Roncal et al., 2011), we recognize 14 well-supported clades within *Geonoma* (Figures 3, 5). We compare our findings with those from the maximum parsimony analysis of 30 morphological traits in the last revision of the group (Henderson, 2011). We use numbers to refer to clades to avoid confusion with the clade names previously used by Roncal et al. (2011) and Henderson (2011).

#### Clade I

This clade comprises a single variable species, *G. maxima* (Poit.) Kunth. It was included by Henderson (2011) in his *G. macrostachys* clade, which corresponds to Clade III in our analysis (Figure 5). *Geonoma maxima* differs, however, from all species of that clade in having a locular epidermis without an operculum, and a higher number of rachillae (4–50 vs. 1–9 rachillae; Henderson, 2011). Henderson (2011) recognized 11 subspecies within this primarily Amazonian lowland species, but the two subspecies with several individuals sampled (*G. maxima* subsp. *camptoneura* (Burret) A.J. Hend. and *G. maxima* subsp. c*helidonura* (Spruce) A.J. Hend) did not form monophyletic groups (Figures 3, 4).

#### Clade II

This clade comprises six species (*G. baculifera* (Poit.) Kunth, *G. calyptrogynoidea* Burret, *G. concinna* Burret, *G. concinnoidea* A.J. Hend, *G. congesta* H, Wendl. ex Spruce, *G. galeanoae* A.J. Hend; Figure 5) that mostly grow in the lowlands of the Chocó region from Costa Rica to north-western Ecuador, with only *G. baculifera* occurring in north-eastern Amazonia and the Guianas. This clade corresponds to Henderson’s (2011) *G. congesta* clade, which is characterized by the prophyll surfaces with close, equal, parallel, and non-dividing ridges. Henderson (2011) did not include *G. galeanoae* in this clade despite also sharing this trait, because its position in his morphology-based maximum parsimony tree was unresolved. Our analysis firmly recovers that species as a member of the clade and as sister to *G. concinna* with strong support (BS of 100, Figure 4 and LPP of 1, Figure 3) emphasizing the taxonomic relevance of this character of the inflorescence bract. Henderson (2011) further recognized two subclades that were also recovered here with good support (BS of 100, Figure 4 and LPP of 1, Figure 3): one including *G. concinna* and *G. concinnoidea* (and *G. galeanoae* in our study), and the other with the remaining species. This latter subclade is characterized by the non-homoplasious character state of staminodial tubes of non-fertilized pistillate flowers projecting and persistent after anthesis (Henderson, 2011).

#### Clade III

The third clade includes nine species [*G. camana* Trail, *G. chlamydostachys* Galeano, *G. chococola* Wess. Boer, *G. macrostachys* Mart., *G. multisecta* (Burret) Burret, *G. oldemannii* Granv., *G. paradoxa* Burret, *G. poiteauana* Kunth, *G. triglochin* Burret; Figure 5] from the Amazonian lowlands and adjacent regions, except for *G. paradoxa* from the Pacific coast of Colombia and Ecuador. It largely corresponds to Henderson’s (2011) *G. macrostachys* clade except for three species (*G. deneversi*, *G. schizocarpa*, and *G. umbraculiformis*) that we did not sequence and *G. maxima*, which we recovered as an independent clade (see above). We recovered two subclades, one composed of *G. macrostachys*, *G. multisecta*, *G. poiteauana*, and *G. oldemannii* and the other of *G. camana, G. chlamydostachys*, *G. chococola, G. paradoxa*, and *G. triglochin* (each clade with bootstrap value of 100, Figure 4 and LPP of 1, Figure 3). These clades did not correspond to the two subgroups recognized by Henderson (2011) in his *G. macrostachys* clade. *Geonoma macrostachys* is one of the morphologically and taxonomically most complex species in the genus, and Henderson (2011) divided it into several morphotypes, some of which behave as sympatric taxa at the local scale (Roncal, 2006; Roncal et al., 2007; Borchsenius et al., 2016). However, the 12 individuals of *G. macrostachys* from four morphotypes that we sampled show that, at larger geographic scales, morphotypes do not cluster into monophyletic groups. The species *G. poiteauna*, which used to be treated as a variety of *G. macrostachys* (Henderson et al., 1995) and subsequently raised at the species level (Henderson, 2011), is recovered here as nested within *G. macrostachys* (Figures 3, 4).

#### Clade IV

This clade includes six species (*G. euspatha* Burret, *G. frontinensis* Burret, *G. interrupta* (Ruiz & Pav.) Mart, *G. pinnatifrons* Willd, *G. santanderensis* Galeano & R. Bernal, *G. simplicifrons* Willd; Figure 5) that largely occur on lower mountain slopes from Costa Rica to Bolivia and northeastern Brazil, as well as in the Antilles. It is essentially identical to Henderson’s (2011) *G. interrupta* clade, except for *G. santanderensis* here recovered as sister to the other five species with strong support (BS of 100, Figure 4 and LPP of 1, Figure 3) whereas it was placed within the *G. stricta* clade in Henderson’s maximum parsimony analysis. Although *G. santanderensis* shares several specific morphological traits with *G. aspidiifolia* Spruce and *G. oligoclona* Trail (such as internodes covered with reddish or brownish scales, rachillae surfaces with spiky, fibrous projections or ridges, staminodial tubes lobed at the apex with the lobes not spreading at anthesis and not acuminate) our phylogenetic analyses reveal that these are homoplasic characters since the species are not closely related. Also, of the two subspecies of *G. interrupta* we sampled, the monophyletic subspecies *rivalis*, endemic of the Central Cordillera in Colombia, is nested within the geographically widespread subspecies *maxima*. The latter is also phenotypically more variable but never includes the rheophytic leaf morphology of subspecies *rivalis*.

#### Clade V

This clade includes three species (*G. bernalii* A. J. Hend, *G. deversa* (Poit.) Kunth, *G. leptospadix* Trail; Figure 5) occuring from Costa Rica to Peru and the Guianas. In Henderson’s (2011) maximum parsimony tree, *G. bernalii* belonged to the *G. lanata* clade, whereas the other two species were placed in an unresolved polytomy. *Geonoma deversa* and *G. leptospadix* are variable and very widespread lowland species which probably hybridize in northeastern Brazil and the Guianas as suggested by the observation of specimens with intermediate morphology (Henderson, 2011) but none of the putative hybrid was sampled here. *Geonoma bernalii* occurs in northern Colombia and was previously identified as *G. leptospadix*. In our phylogenetic tree, *G. leptospadix* appears as sister to a group formed by *G. bernalii* and *G. deversa.*

#### Clade VI

This clade includes two morphologically very similar species (*G. aspidiifolia* and *G. oligoclona*; Figure 5) from Amazonia and the Guianan highlands. In fact, out of the two specimens of *G. oligoclona*, one is recovered more closely related to the single specimen of *G. aspidiifolia* (with BS of 74, Figure 4 and LPP of 1, Figure 3). In the absence of additional individuals of *G. aspidiifolia*, it is premature to conclude whether they actually represent a single variable species or two closely related species. In Henderson’s (2011) maximum parsimony tree these two species were placed within the *G. stricta* clade and were closely related to *G. santanderensis.*

#### Clade VII

This clade includes four species (*G. elegans* Mart., *G. pauciflora* Mart., *G. pohliana* Mart., and *G. schottiana* Mart.; Figure 5) from the Brazilian Atlantic Forest and the Cerrado. It corresponds to Henderson’s (2011) *G. schottiana* clade and to Roncal et al. (2011) Brazilian Cerrado + Mata Atlantica clade. It appears that this small radiation of south-eastern Brazilian species resulted from a single colonization event that gave rise to four species with considerable inter- and intraspecific variation. In our phylogeny, the two individuals of *G. schottiana* were recovered as nested within *G. pohliana*, indicating that similarly to what is observed in other species complexes, morphological taxa are not always underpinned by strong genetic differentiation. *Geonoma pohliana* is an extremely variable species complex which Henderson (2011) subdivided into 11 subspecies. The three subspecies sampled in our analysis appeared to be randomly mixed. The other two Brazilian species *G. elegans* and *G. pauciflora*, were recovered paraphyletic (Figures 3, 4).

#### Clade VIII

This clade includes two morphologically similar endemic species from Venezuela (*G. spinescens* H. Wendl. ex Burret and *G. braunii* (Stauffer) A.J. Hend; Figure 5). Little DNA was obtained from the three herbarium samples and in fact *G. braunii* was recovered as sister taxa to clades VIII-XVI in the two ASTRAL analyses (Figure 3). However, we believe that this is caused by the lack of DNA sequences for *G. braunii.* Therefore, despite this uncertainty, we decided to follow the topology of the concatenated analysis and treat the two species as part of a single clade because it is coherent with the fact that *G. braunii* used to be considered a variety of *G. spinescens* (Stauffer, 1997). Henderson (2011) treated them as distinct species based on the flower pits alternately arranged in the former and spirally arranged in the latter. The two species were included within Henderson’s (2011) *G. lanata* clade. In our concatenated analysis, *G. spinescens* appears paraphyletic because one of the two samples of *G. spinescens* was found sister to *G. braunii* (with bootstrap support of 99, Figure 4) but given the low amount of sequence data for *G. braunii* we can not advocate either of the two possible taxonomic treatment.

#### Clade IX

This clade includes two species (*G. hollinensis* A.J. Hend, Borchs & Balslev, and *G. triandra* (Burret) Wess. Boer; Figure 5) that are distributed from Panama to Ecuador and occur at similar elevations. The geographic distribution of these sister species (*G. hollinensis* restricted to north-eastern Ecuador and *G. triandra* found from north-western Ecuador to southern Panama) suggest that vicariance was involved in their divergence. Both species have staminate flowers with three stamens, and were segregated as subgenus *Kalbreyera* by Wessels Boer (1968). Henderson (2011) placed them together with *G. occidentalis* in the *G. triandra* clade but we cannot confirm this placement since *G. occidentalis* was not included in our study.

#### Clade X

This clade includes samples of five species (*G. lehmannii* Dammer ex Burret, *G. orbignyana* Mart., *G. talamancana* Grayum, *G. trigona* (Ruiz & Pav.) A.H. Gentry and *G. undata*; Figure 5) that occur at high elevations from Mexico to Bolivia, also reaching the Lesser Antilles, plus *G. fosteri* A.J. Hend. It is largely congruent with the *G. undata* clade of Henderson (2011) and the Andes + Central American Mountains clade of Roncal et al. (2010, 2011). Except for *G. fosteri*, the species of this clade share the character state of apiculate and lobed proximal lips of the flower pits. This is one of the taxonomically most complex groups of the genus, and species delimitation has been handled differently over time (Henderson et al., 1995; Henderson, 2011). In our study, the samples assigned to the widespread species *G. orbignyana* and *G. undata* were not recovered as phylogenetically independent lineages (Figures 3, 4). Rather than clustering according to taxonomic delimitations, specimens of these two species grouped with strong support by geographic location, forming two main clades (each with BS of 100, Figure 4 and LPP of 1, Figure 3): (a) a central American – north Andean clade composed of specimens from Mexico, Costa-Rica, Panama, the Caribbean, and Colombia; and (b) a north Andean – central Andean clade composed of specimens from Ecuador, Peru, and Bolivia. However, in addition to these two subclades, there was also a separate group at the base of the clade, comprising three specimens from Ecuador and Bolivia in the coalescent analysis (with LPP of 1, Figure 3) or these three specimens plus *G. trigona* in the concatenated analysis (with BS of 63, Figure 4). The samples of *G. lehmannii* subsp. *corrugata* A.J. Hend. and *G. talamancana*, both occurring in Central America, were nested within the central American – north Andean clade with strong support (BS of 100, Figure 4 and LPP of 1, Figure 3). As these two species have resembling morphologies and similar high elevation habitats to *G. undata* and *G. orbignyana*, they may represent locally divergent populations of this broad species complex, in which a novel phenotype has been fixed (e.g., the thick corrugated leaves and the well-developed peduncle in *G. lehmannii* subsp. *corrugata)* although it is not underpinned by strong genetic isolation. Another similar case is *G. trigona*, which, together with *G. fosteri* is recovered as sister to the rest of Clade X in the coalescent analysis (with LPP of 0.8, Figure 3). The placement of *G. fosteri* is intriguing because it is morphologically clearly different from the rest of the species in this clade, even though it occurs in the same habitat. Although high-quality DNA was recovered for this sample and the identification of the living specimen from which it was collected was double-checked, we cannot rule out contamination during laboratory work to explain this surprising result.

#### Clade XI

This clade includes five species (*G. cuneata* H. Wendl. ex Spruce, *G. lanata* A.J. Hend, Borchs & Balslev, *G. laxiflora* Mart., *G. stricta* (Poit.) Kunth, *G. tenuissima* H.E. Moore, Figure 5) that Henderson (2011) placed in several distinct clades (*G. cuneata*, *G. lanata*, and *G. stricta* clades). These five species were part of an unresolved clade recovered in Roncal et al. (2012). *Geonoma stricta* and *G. cuneata* are two species complexes whereas the three remaining species of this clade have rather low variability (Henderson, 2011). Henderson (2011) recognized nine subspecies in *G. stricta*, with the most widespread and morphologically variable, subspecies *arundinacea*, further divided into eight morphotypes. Of the three subspecies included in our analysis, subspecies *stricta* and *montana* were nested within *arundinacea*. For the latter, the two morphotypes sampled (*trailii* and *pycnostachys*) were paraphyletic. In *G. cuneata*, nine subspecies were recognized and the most widespread and morphologically variable, subspecies *cuneata* was divided into 13 morphotypes (Henderson, 2011). We did not sample enough individuals of the different subspecies of *G. cuneata* to comment on their recognition using phylogenomics, except that subspecies *guanacastensis* was nested within subspecies *cuneata*. Species of this clade are mostly distributed in lowland and lower montane forests with *G. cuneata* distributed from Nicaragua to Ecuador, *G. lanata* and *G. tenuissima* occurring mostly on the western Andean slopes, *G. laxiflora* in western Amazonia, and the widespread *G. stricta* overlapping the previous four ranges and reaching central Amazonia and the Guyanas. Although these species are morphologically quite different from each other, which explains why Henderson (2011) recovered them in different clades, they commonly have cane-like stems with yellowish and smooth internodes.

#### Clade XII

This clade includes a single species, *G. divisa* H.E. Moore (Figure 5), which is endemic to northwestern Colombia. Henderson (2011) placed this species in his *G. stricta* clade, alongside *G. longivaginata* H.Wendl. ex Spruce and *G. ferruginea* H. Wendl. ex Spruce, but this relationship was not supported at all in our analyses. Morphologically, *G. divisa* differs from these two species in its tricussately arranged, closely spaced flower pits (Henderson, 2011).

#### Clade XIII

This clade includes two species (*G. brongniartii* Mart., *G. poeppigiana* Mart.; Figure 5) that occur from Colombia to Bolivia. These closely related species are variable and their separation has long been debated (Henderson, 2011). In our phylogenetic tree, these two morphological taxa are mixed together within a single clade rather than forming distinct monophyletic groups (Figures 3, 4). Although we can not rule out sample misidentification, this result could also be explained by alternative hypotheses. Indeed, the geographic distributions of these two species overlap, with *G. poeppigiana* having a smaller range than *G. brongniartii*, and Henderson (2011) reported putative hybrids between them in central Peru. All specimens of *G. poeppigiana* included in our phylogeny come from Peru and so do most specimens of *G. brongniartii*. If the two species indeed hybridize in the Peruvian area where they co-occur, this may explain why they are mixed in our phylogenetic tree. Alternatively, these samples could represent a single widely variable species (see below).

#### Clade XIV

This clade includes eight species (*G. brenesii* Grayum, *G. epetiolata* H.E. Moore, *G. ferruginea*, *G. hugonis* Grayum & de Nevers, *G. longivaginata*, *G. monospatha* de Nevers, *G. mooreana* de Nevers & Grayum, *G. scoparia* Grayum & de Nevers; Figure 5) from Costa Rica and Panama. It corresponds to Roncal et al.’s (2011) central American clade, whereas in Henderson’s (2011) tree these species were placed in three different clades (namely the *G. cuneata*, *G. lanata*, and *G. stricta* clades). The three samples of *G. monospatha* from Costa Rica are placed far apart from the two Panamanian samples in our phylogenetic tree (with BS of 100, Figure 4 and LPP of 1, Figure 3). These populations are geographically disjunct, with a gap of several hundred kilometers between them. Furthermore, the Costa Rican specimens have smaller leaves and inflorescences and thrive at higher elevations (mean elevations 1750 m vs. 837 m). All of this suggests that the Costa Rican population may better be treated as a distinct species. Similarly, the two samples of the Panamanian *G. longivaginata* subspecies *copensis* A. J. Hend. are more closely related to the two Panamanian samples of *G. monospatha* (with BS of 100, Figure 4 and LPP of 1, Figure 3) than to the individuals of *G. longivaginata* from Costa Rica, suggesting that this subspecies may, in fact, also better be treated as a distinct species.

### Comparison With Other Phylogenetic Reconstructions

In the latest revision of *Geonoma*, Henderson (2011) conducted a maximum parsimony phylogenetic analysis of all the species of the genus based on 30 qualitative morphological characters. We used his species-level taxonomy to name our taxa and tried to apply his clade definition to the phylogenetic tree we obtained. We found that many of his clades were supported by our study, although often with the exclusion or inclusion of a few species. For instance, his *G. cuneata* clade (minus *G. cuneata*) corresponds to our Clade XIV, his *G. macrostachys* clade (minus *G. maxima*) corresponds to our Clade III, his *G. schottiana* clade corresponds to our Clade VII, his *G. undata* clade (plus *G. fosteri*) corresponds to our Clade X, his *G. congesta* clade (plus *G. galeanoae*) to our Clade II, and his *G. interrupta* clade (plus *G. santaderensis*) to our Clade IV. In contrast, Henderson’s *G. lanata* and *G. stricta* clades are not supported at all by our analyses, suggesting that these clades were defined by homoplasic morphological characters with limited phylogenetic information. These characters included rachillae surfaces with spiky, fibrous projections or ridges for the *G. stricta* clade, and filiform rachillae with extended narrowed sections between the alternately arranged flower pits for the *G. lanata* clade. Furthermore, the relationships between the clades as recovered by Henderson (2011) are in strong disagreement with the topology obtained from molecular data, indicating that morphology provides little insight on the deep phylogenetic relationships within the genus.

The first molecular phylogeny of *Geonoma* was based on 20 species and two markers (Roncal et al., 2005). It was later extended to three genes and 43 species (Roncal et al., 2010, 2011, 2012). Using an extended sampling of 57 species and 795 gene regions, our study confirmed many of the findings of these studies for the phylogenetic relationships at intermediate levels of divergence. For instance, our Clades I-III, which together are sister to the other 11 clades, correspond to Roncal’s (2011) Amazon clade, which was also recovered as sister to the remainder of the genus. Furthermore, the internal arrangements of the species in this group are also largely congruent, with *G. maxima* sister to the remainder of the species in the Amazon clade, and *G. calyptrogynoidea* and *G. congesta* (our Clade II) sister to the remainder of the species (our Clade III), although *G. baculifera* and *G. concinna* were recovered by Roncal et al. (2010) to be more closely related to species in our Clade III than they were to species in Clade II, where we placed them. Likewise, Roncal et al.’s (2011) Brazilian Cerrado + Mata Atlantica, Andes + Central American Mountains, and Central America clades were also recovered in our phylogenetic tree and the relative arrangements of these clades are overall similar between both studies. In general, previously unresolved phylogenetic relationships were resolved with strong support in our analyses.

### Species Delimitation

Henderson (2011) used a statistical approach in which species delimitation was based on the results of a clustering analysis of qualitative morphological characters. The congruence between the results of this method and those of our phylogenetic analysis is striking. Indeed, the majority of species and species complexes for which we included several samples were recovered as monophyletic units in our phylogenetic trees. Furthermore, morphological variation seemed to be correlated to genetic divergence, as indicated by the longer branches of widely variable species in the maximum likelihood tree compared to species with smaller geographic ranges and less variable phenotypes (Figure 4). Although further sampling would be necessary in order to include the full range of variability of some widely distributed species, our study nevertheless supports the validity of the characters used by Henderson (2011) to define species boundaries. Conversely, even though not all our samples had identification at the intraspecific level, our results indicate that the intraspecific divisions as subspecies, varieties or morphotypes generally do not match the genetic clusters. These intraspecific taxa were defined by Henderson (2011) usually based on a few, often variable characters such as the degree of division in the inflorescences. Only in *G. pinnatifrons* are the subspecies, which were delimited based on geographical distribution, supported by the tree topology (Figures 3, 4). In other species complexes (e.g., in *G. cuneata*, *G. maxima, G. stricta*, *G. macrostachys*, and *G. pohliana)*, intraspecific taxa are not recovered as monophyletic (Figures 3, 4). Furthermore, while our phylogenetic tree revealed a broad North-South differentiation within the mixed *G. orbignyana – G. undata* group, in general there seems to be no geographic clustering of individuals, especially for widespread Amazonian taxa such as *G. macrostachys*, *G. maxima* or *G. stricta*. Various hypotheses have been proposed to explain this kind of chaotic intraspecific phenotypic variation, such as population contraction and expansion during Pleistocene climatic oscillations (Cronk, 1998), rapid dispersal followed by selection (Cronk, 1998), or niche divergence induced by forest heterogeneity (Henderson, 2011). From a genetic perspective, incomplete lineage sorting or hybridization are commonly invoked to explain the widespread occurrence of plant species complexes similar to those found in *Geonoma* (Bacon et al., 2012b; Pinheiro et al., 2018). Although our results pointed to high level of gene tree incongruence in the species complexes of *Geonoma* (Figure 3), likely due to incomplete lineage sorting, it is beyond the scope of our study to test for any of these underlying mechanisms. Further phylogeographic or population genetic studies are needed to understand the origin of the discrepancy between morphological and genetic data in this group.

From a systematic point of view, the remaining issue to be addressed is the taxonomic status of the several non-monophyletic species that were identified by our analysis. First, there are two cases where two species were recovered mixed within a single clade (*G. brongniartii* with *G. poeppigiana* and *G. orbignyana* with *G. undata*). Second, there are several

instances of geographically restricted species (e.g., *G. lehmanii, G. poiteauana, G. talamancana*, and *G. trigona*) which were found to be nested within more widely distributed species, making the latter paraphyletic. For taxonomic classification, there are two fundamentally different approaches to deal with such situations. On one hand, under a lineage species concept, which requires species monophyly, the phylogenetically intermixed “species” of *Geonoma* would be considered to represent single variable species as was done in other similar cases in plants (Bennett et al., 2008; Barbosa et al., 2012). Applying this approach would entail a reduction in the number of recognized species within *Geonoma*. On the other hand, some authors have stressed that at the species level paraphyly is not an issue because it is considered to be the natural output of, for example, peripatric speciation or speciation via polyploidy which are common phenomena in plants (Rieseberg and Brouillet, 1994; Crisp and Chandler, 1996). Likewise, the pattern of small-range species nested within large-range paraphyletic species has been suggested to be common in rainforest trees with widespread distribution for which coalescence times are long due to large population size and extensive gene flow (Pennington and Lavin, 2016). Non-monophyletic species can also arise from hybridization, which is widespread in plants (Whitney et al., 2010). Under a genic species concept, species cohesion may be determined by a small number of genes, thus allowing gene flow between species without calling species identity into question (Wu, 2001; Lexer and Widmer, 2008). Therefore, adopting a species concept which places emphasis on the phenotype would result in treating morphologically divergent entities as separate species even if they do no represent evolutionary independent lineages (Freudenstein et al., 2016). From this point of view, the number of species in *Geonoma* would remain similar to that proposed by Henderson (2011). In the end, how the phylogenetic information presented here is translated into a taxonomic classification is to a certain degree a matter of personal preference, with different researchers favoring different aspects. Thus, some may emphasize morphological or genetic similarities while others would focus on differences, some place more importance on the ability to diagnose taxa while others prioritize evolutionary independence, and so on. We refrain from proposing taxonomic decisions based on our results, since this would require a full assessment of genetic, morphological, and ecological evidence.

## Conclusion

By employing a large novel set of molecular markers, we were able to clarify both deep and shallow phylogenetic relationships within the tribe Geonomateae including for *Geonoma*, one of the largest and taxonomically most challenging Neotropical palm genera. The remaining poorly supported phylogenetic relationships do not reflect a lack of informative genetic data but are rather caused by a high level of gene tree incongruence, as shown by the coalescent analysis. Our phylogenetic analyses revealed two cryptic species of *Geonoma* in Central America, which will have to be described in further taxonomic work. The intraspecific sampling confirmed in most cases the validity of the taxonomic delimitation of species proposed by Henderson (2011),

even for those with extensive phenotypic variability such as *G. cuneata*, *G. interrupta*, *G. maxima*, *G. pinnatifrons*, *G. macrostachys*, or *G. stricta.* However, we also pointed to several cases where the morphological delimitations do not reflect the genetic clusters, such as the internal delimitations of widely variable species complexes, the clustering of rare endemic species within broader species complexes, or the mixing of two species complexes. These groups that do not show clear genetic boundaries between morphologically recognized taxa remain the main challenge in the systematics of *Geonoma.* Ultimately, the number of species recognized in *Geonoma* depends on the species concept one endorses.

Studies at the population level are needed to understand whether the decoupling between morphological and genetic variation in the species complexes is the result of ongoing speciation with gene flow or from secondary contact and hybridization between previously diverged taxa. Although the impossibility of summarizing morphological variation of these groups into a coherent classification scheme may seem frustrating from a taxonomic point of view, we argue that it represents a unique opportunity to better understand the build-up of Neotropical plant diversity. Indeed, species complexes are common in plants and are gaining attention as model groups to study the underlying factors of plant speciation (Pinheiro et al., 2018). In this context, the set of baits recently developed by de La Harpe et al. (2019) will therefore be a useful tool to carry out specific population levels studies.

With this in mind, we provided the baits for a selection including 20% of the most informative genes from the kit developed by de La Harpe et al. (2019) by assessing their phylogenetic informativeness across three Arecaceae subfamilies. We predict that these baits should work over a wide evolutionary timescale in the Arecaceae and will therefore benefit the whole field of palm phylogenomics. Indeed, the smaller size of this kit will make it accordingly more affordable and will reduce the computation time of post-sequencing bioinformatic analyses while maximizing the phylogenetic informativeness at deep and shallow scales across the Arecaceae family. Hence, we believe that it has the potential to be an essential tool in the search toward a complete species-level phylogeny of the Arecaceae family.

## Data Availability

Targeted sequence reads generated as part of this manuscript are available in NCBI (BioProject PRJNA541164). The list of the 795 selected genes and their corresponding bait sequences in fasta format as well as all gene trees and species trees were deposited in Zenodo (deposit number 2594808).

## Author Contributions

NS, MK, and CL designed the study. MPa led the sequencing experiment and performed the post-sequencing bioinformatics analyses. MdLH, OL, TM-M, and MPa did the labwork. OL and

DK performed the phylogenetic analyses. CB, HB, FB, AC, TC, MPe, JuR, MS, FS, CL, MK, and NS were part of the Geonoma Consortium set up to perform this study. OL led the writing with significant contributions from all co-authors, in particular MK, IO, and NS. All co-authors commented and agreed on the last version of the manuscript.

## Conflict of Interest Statement

The authors declare that the research was conducted in the absence of any commercial or financial relationships that could be construed as a potential conflict of interest.

## Footnotes

1 https://doi.org/10.5061/dryad.3v9v238

2 http://broadinstitute.github.io/picard

3 https://github.com/JinfengChen/vcf-tab-to-fasta

4 https://github.com/sidonieB/scripts/blob/master/GetQpiechartsFrom ASTRAL.py

## References

[B1] Abadi S. Azouri D. Pupko T. Mayrose I. (2019). Model selection may not be a mandatory step for phylogeny reconstruction. *Nat. Commun.* 10:934. 10.1038/s41467-019-08822-w 30804347 PMC6389923

[B2] Al-Mssallem I. S. Hu S. Zhang X. Lin Q. Liu W. Tan J. (2013). Genome sequence of the date palm *Phoenix dactylifera* L. *Nat. Commun.* 4:2274. 10.1038/ncomms3274 23917264 PMC3741641

[B3] Bacon C. D. Baker W. J. Simmons M. P. (2012a). Miocene dispersal drives island radiations in the palm tribe Trachycarpeae (Arecaceae). *Syst. Biol.* 61 426–442. 10.1093/sysbio/syr123 22223444

[B4] Bacon C. D. McKenna M. J. Simmons M. P. Wagner W. L. (2012b). Evaluating multiple criteria for species delimitation: an empirical example using Hawaiian palms (Arecaceae: *Pritchardia*). *BMC Evol. Biol.* 12:23. 10.1186/1471-2148-12-23 22353848 PMC3356231

[B5] Bacon C. D. Look S. L. Gutiérrez-Pinto N. Antonelli A. Tan H. T. Kumar P. P. (2016a). Species limits, geographical distribution and genetic diversity in *Johannesteijsmannia* (Arecaceae). *Bot. J. Linn. Soc.* 182 318–347. 10.1111/boj.12470

[B6] Bacon C. D. Velásquez-Puentes F. Flórez-Rodríguez A. Balslev H. Galeano G. Bernal R. (2016b). Phylogenetics of Iriarteeae (Arecaceae), cross-Andean disjunctions and convergence of clustered infructescence morphology in *Wettinia*. *Bot. J. Linn. Soc.* 182 272–286. 10.1111/boj.12421

[B7] Bacon C. D. Moraes M. Jaramillo C. Antonelli A. (2017). Endemic palm species shed light on habitat shifts and the assembly of the Cerrado and Restinga floras. *Mol. Phylogenet. Evol.* 110 127–133. 10.1016/j.ympev.2017.03.013 28288942

[B8] Baker W. J. Asmussen C. B. Barrow S. C. Dransfield J. Hedderson T. A. (1999). A phylogenetic study of the palm family (Palmae) based on chloroplast DNA sequences from the trnL - trnF region. *Plant Syst. Evol.* 219 111–126. 10.1007/BF01090303

[B9] Baker W. J. Couvreur T. L. P. (2013). Global biogeography and diversification of palms sheds light on the evolution of tropical lineages. II. Diversification history and origin of regional assemblages. *J. Biogeogr.* 40 286–298. 10.1111/j.1365-2699.2012.02794.x

[B10] Baker W. J. Dransfield J. (2016). Beyond *Genera Palmarum*: progress and prospects in palm systematics. *Bot. J. Linn. Soc.* 182 207–233. 10.1111/boj.12401

[B11] Baker W. J. Norup M. V. Clarkson J. J. Couvreur T. L. P. Dowe J. L. Lewis C. E. (2011). Phylogenetic relationships among arecoid palms (Arecaceae: Arecoideae). *Ann. Bot.* 108 1417–1432. 10.1093/aob/mcr020 21325340 PMC3219489

[B12] Baker W. J. Savolainen V. Asmussen-Lange C. B. Chase M. W. Dransfield J. Forest F. (2009). Complete generic-level phylogenetic analyses of palms (arecaceae) with comparisons of supertree and supermatrix approaches. *Syst. Biol.* 58 240–256. 10.1093/sysbio/syp021 20525581

[B13] Balslev H. Copete J. Pedersen D. Bernal R. Galeano G. Duque Á, et al. (2017). “Palm diversity and abundance in the Colombian Amazon,” in *Forest structure, function and dynamics in Western Amazonia*, ed. Myster R. W. (London: John Wiley & Sons Ltd.).

[B14] Balslev H. Laumark P. Pedersen D. Grández C. (2016). Tropical rainforest palm communities in Madre de Dios in Amazonian Peru. *Rev. Peru. Biol.* 23 3–12.

[B15] Barbosa A. R. Fiorini C. F. Silva-Pereira V. Mello-Silva R. Borba E. L. (2012). Geographical genetic structuring and phenotypic variation in the *Vellozia hirsuta* (Velloziaceae) ochlospecies complex. *Am. J. Bot.* 99 1477–1488. 10.3732/ajb.1200070 22889618

[B16] Barrett C. Sinn B. King L. Medina J. Bacon C. Lahmeyer S. (2018). Phylogenomics, biogeography, and evolution in the American palm genus *Brahea*. *bioRxiv* 467779. 10.1101/467779

[B17] Barrett C. F. Bacon C. D. Antonelli A. Hofmann T. (2016). An introduction to plant phylogenomics with a focus on palms. *Bot. J. Linn. Soc.* 182 234–255. 10.1111/boj.12399

[B18] Bennett J. R. Wood J. R. I. Scotland R. W. (2008). Uncorrelated variation in widespread species: species delimitation in *Strobilanthes echinata* Nees (Acanthaceae). *Bot. J. Linn. Soc.* 156 131–141. 10.1111/j.1095-8339.2007.00756.x

[B19] Blom M. P. K. Bragg J. G. Potter S. Moritz C. (2017). Accounting for uncertainty in gene tree estimation: summary-coalescent species tree inference in a challenging radiation of Australian lizards. *Syst. Biol.* 66 352–366. 10.1093/sysbio/syw089 28039387

[B20] Borchsenius F. (2002). Staggered flowering in four sympatric varieties of *Geonoma cuneata* (Palmae). *Biotropica* 34 603–606. 10.1111/j.1744-7429.2002.tb00580.x

[B21] Borchsenius F. Lozada T. Knudsen J. T. (2016). Reproductive isolation of sympatric forms of the understorey palm *Geonoma macrostachys* in western Amazonia. *Bot. J. Linn. Soc.* 182 398–410. 10.1111/boj.12428

[B22] Chazdon R. L. (1991). Plant size and Form in the understory palm genus *Geonoma*: are species variations on a theme? *Am. J. Bot.* 78 680–694. 10.1002/j.1537-2197.1991.tb12592.x

[B23] Chazdon R. L. (1992). Patterns of growth and reproduction of *Geonoma congesta*, a clustered understory palm. *Biotropica* 24 43–51.

[B24] Chernomor O. von Haeseler A. Minh B. Q. (2016). Terrace aware data structure for phylogenomic inference from supermatrices. *Syst. Biol.* 65 997–1008. 10.1093/sysbio/syw037 27121966 PMC5066062

[B25] Comer J. R. Zomlefer W. B. Barrett C. F. Davis J. I. Stevenson D. W. Heyduk K. (2015). Resolving relationships within the palm subfamily Arecoideae (Arecaceae) using plastid sequences derived from next-generation sequencing. *Am. J. Bot.* 102 888–899. 10.3732/ajb.1500057 26101415

[B26] Comer J. R. Zomlefer W. B. Barrett C. F. Stevenson D. W. Heyduk K. Leebens-Mack J. H. (2016). Nuclear phylogenomics of the palm subfamily Arecoideae (Arecaceae). *Mol. Phylogenet. Evol.* 97 32–42. 10.1016/j.ympev.2015.12.015 26748268

[B27] Couvreur T. L. P. Forest F. Baker W. J. (2011). Origin and global diversification patterns of tropical rain forests: inferences from a complete genus-level phylogeny of palms. *BMC Biol.* 9:44. 10.1186/1741-7007-9-44 21679405 PMC3142250

[B28] Crisp M. D. Chandler G. T. (1996). Paraphyletic species. *Telopea* 6 813–844. 10.7751/telopea19963037

[B29] Cronk Q. (1998). “The ochlospecies concept,” in *Chorology, Taxonomy and Ecology of the Floras of Africa and Madagascar*, eds Huxley C. R. Lock J. M. Cutler D. F. (Kew: Royal Botanic Gardens), 155–170.

[B30] Cuenca A. Asmussen-Lange C. B. Borchsenius F. (2008). A dated phylogeny of the palm tribe Chamaedoreeae supports Eocene dispersal between Africa, North and South America. *Mol. Phylogenet. Evol.* 46 760–775. 10.1016/j.ympev.2007.10.010 18357644

[B31] Danecek P. Auton A. Abecasis G. Albers C. A. Banks E. DePristo M. A. (2011). The variant call format and VCFtools. *Bioinformatics* 27 2156–2158. 10.1093/bioinformatics/btr330 21653522 PMC3137218

[B32] de La Harpe M. Hess J. Loiseau O. Salamin N. Lexer C. Paris M. (2019). A dedicated target capture approach reveals variable genetic markers across micro-and macro-evolutionary time scales in palms. *Mol. Ecol. Resour.* 19 221–234. 10.1111/1755-0998.12945 30240120

[B33] Dransfield J. Uhl N. W. Asmussen C. B. Baker W. J. Harley M. M. Lewis C. E. (2008). *Genera Palmarum: The Evolution and Classifi Cation of Palms*, 2nd Edn Kew: Royal Botanical Gardens.

[B34] Faircloth B. C. Chang J. Alfaro M. E. (2012). TAPIR enables high-throughput estimation and comparison of phylogenetic informativeness using locus-specific substitution models. *arXiv preprint* arXiv:1202.1215.

[B35] Freudenstein J. V. Broe M. B. Folk R. A. Sinn B. T. (2016). Biodiversity and the species concept—lineages are not enough. *Syst. Biol.* 66 644–656. 10.1093/sysbio/syw098 27798406

[B36] Gruca M. Blach-Overgaard A. Balslev H. (2015). African palm ethno-medicine. *J. Ethnopharmacol.* 165 227–237. 10.1016/j.jep.2015.02.050 25749399

[B37] Henderson A. (2002). *Evolution and Ecology of Palms.* New York, NY: New York Botanical Garden Press.

[B38] Henderson A. (2005). A multivariate study of *Calyptrogyne* (Palmae). *Syst. Bot.* 30 60–83. 10.1600/0363644053661913

[B39] Henderson A. (2011). A revision of *Geonoma* (Arecaceae). *Phytotaxa* 17 1–271.

[B40] Henderson A. (2012). A revision of *Pholidostachys* (Arecaceae). *Phytotaxa* 43 1–48.

[B41] Henderson A. Galeano G. Bernal R. (1995). *A Field Guide to the Palms of the Americas.* Princeton, NJ: Princeton University Press.

[B42] Henderson A. Martins R. (2002). Classification of specimens in the *Geonoma stricta* (Palmae) complex: the problem of leaf size and shape. *Brittonia* 54 202–212. 10.1663/0007-196x(2002)054[0202:cositg]2.0.co;2

[B43] Henderson A. Villalba I. (2013). A revision of *Welfia* (Arecaceae). *Phytotaxa* 119 33–44. 10.11646/phytotaxa.119.1.3

[B44] Heyduk K. Trapnell D. W. Barrett C. F. Leebens-mack J. I. M. (2015). Phylogenomic analyses of species relationships in the genus *Sabal* (Arecaceae) using targeted sequence capture. *Biol. J. Linn. Soc.* 117 106–120. 10.1111/bij.12551

[B45] Hoang D. T. Chernomor O. von Haeseler A. Minh B. Q. Vinh L. S. (2018). UFBoot2: improving the ultrafast bootstrap approximation. *Mol. Biol. Evol.* 35 518–522. 10.1093/molbev/msx281 29077904 PMC5850222

[B46] Hoff M. Orf S. Riehm B. Darriba D. Stamatakis A. (2016). Does the choice of nucleotide substitution models matter topologically? *BMC Bioinformatics* 17:143. 10.1186/s12859-016-0985-x 27009141 PMC4806516

[B47] Kircher M. Sawyer S. Meyer M. (2011). Double indexing overcomes inaccuracies in multiplex sequencing on the Illumina platform. *Nucleic Acids Res.* 40:e3. 10.1093/nar/gkr771 22021376 PMC3245947

[B48] Knudsen J. T. Andersson S. Bergman P. Bergman P. Knudsen J. T. (1998). Floral scent attraction in *Geonoma macrostachys*, an understorey palm of the Amazonian rain forest. *Oikos* 85 409–418.

[B49] Langmead B. Salzberg S. L. (2012). Fast gapped-read alignment with Bowtie 2. *Nat. Methods* 9 357–359. 10.1038/nmeth.1923 22388286 PMC3322381

[B50] Lexer C. Widmer A. (2008). The genic view of plant speciation: recent progress and emerging questions. *Philos. Trans. R. Soc. Lond. B Biol. Sci.* 363 3023–3036. 10.1098/rstb.2008.0078 18579476 PMC2607315

[B51] Ley-lopez J. M. Avalos G. (2017). Propagation of the palm flora in a lowland tropical rainforest in Costa Rica: fruit collection and germination patterns. *Trop. Conserv. Sci.* 10 1–12. 10.1177/1940082917740703

[B52] Liu L. Xi Z. Wu S. Davis C. C. Edwards S. V. (2015). Estimating phylogenetic trees from genome-scale data. *Ann. N. Y. Acad. Sci.* 1360 36–53. 10.1111/nyas.12747 25873435

[B53] Liu L. Yu L. Kubatko L. Pearl D. K. Edwards S. V. (2009). Coalescent methods for estimating phylogenetic trees. *Mol. Phylogenet. Evol.* 53 320–328. 10.1016/j.ympev.2009.05.033 19501178

[B54] Macia M. J. Armesilla P. J. Cámara-Leret R. Paniagua-Zambrana N. Villalba S. Balslev H. (2011). Palm uses in northwestern South America: a quantitative review. *Bot. Rev.* 77 462–570. 10.1007/s12229-011-9086-8

[B55] Mandel J. R. Barker M. S. Bayer R. J. Dikow R. B. Gao T. Jones K. E. (2017). The compositae tree of life in the age of phylogenomics. *J. Syst. Evol.* 55 405–410. 10.1111/jse.12265

[B56] McKenna A. Hanna M. Banks E. Sivachenko A. Cibulskis K. Kernytsky A. (2010). The genome analysis toolkit: a MapReduce framework for analyzing next-generation DNA sequencing data. *Genome Res.* 20 1297–1303. 10.1101/gr.107524.110 20644199 PMC2928508

[B57] Meerow A. W. Noblick L. Salas-Leiva D. E. Sanchez V. Francisco-Ortega J. Jestrow B. (2015). Phylogeny and historical biogeography of the cocosoid palms (Arecaceae, Arecoideae, Cocoseae) inferred from sequences of six WRKY gene family loci. *Cladistics* 31 509–534. 10.1111/cla.12100 34772273

[B58] Meyer M. Kircher M. (2010). Illumina sequencing library preparation for highly multiplexed target capture and sequencing. *Cold Spring Harb. Protoc.* 2010:db.rot5448. 10.1101/pdb.prot5448 20516186

[B59] Mirarab S. Reaz R. Bayzid M. S. Zimmermann T. Swenson M. S. Warnow T. (2014). ASTRAL: genome-scale coalescent-based species tree estimation. *Bioinformatics* 30 i541–i548. 10.1093/bioinformatics/btu462 25161245 PMC4147915

[B60] Mirarab S. Warnow T. (2015). ASTRAL-II: coalescent-based species tree estimation with many hundreds of taxa and thousands of genes. *Bioinformatics* 31 i44–i52. 10.1093/bioinformatics/btv234 26072508 PMC4765870

[B61] Mitchell N. Lewis P. O. Moriarty Lemmon E. Lemmon A. R. Holsinger K. E. (2017). Anchored phylogenomics improves the resolution of evolutionary relationships in the rapid radiation of *Protea* L. *Am. J. Bot.* 104 102–115. 10.3732/ajb.1600227 28104589

[B62] Muscarella R. Bacon C. D. Faurby S. Antonelli A. Kristiansen S. M. Svenning J.-C. (2018). Soil fertility and flood regime are correlated with phylogenetic structure of Amazonian palm communities. *Ann. Bot.* 123 641–655. 10.1093/aob/mcy196 30395146 PMC6417467

[B63] Nguyen L.-T. Schmidt H. A. von Haeseler A. Minh B. Q. (2015). IQ-TREE: a fast and effective stochastic algorithm for estimating maximum-likelihood phylogenies. *Mol. Biol. Evol.* 32 268–274. 10.1093/molbev/msu300 25371430 PMC4271533

[B64] Nicholls J. A. Pennington R. T. Koenen E. J. M. Hughes C. E. Hearn J. Bunnefeld L. (2015). Using targeted enrichment of nuclear genes to increase phylogenetic resolution in the neotropical rain forest genus *Inga* (Leguminosae: Mimosoideae). *Front. Plant Sci.* 6:710. 10.3389/fpls.2015.00710 26442024 PMC4584976

[B65] Pennington R. T. Lavin M. (2016). The contrasting nature of woody plant species in different neotropical forest biomes reflects differences in ecological stability. *New Phytol.* 210 25–37. 10.1111/nph.13724 26558891

[B66] Pinheiro F. Dantas-queiroz M. V. Palma-silva C. (2018). Plant species complexes as models to understand speciation and evolution: a review of South American studies. *Crit. Rev. Plant Sci.* 37 54–80. 10.1080/07352689.2018.1471565

[B67] Pizo M. A. Almeida-Neto M. (2009). Determinants of fruit removal in *Geonoma pauciflora*, an understory palm of neotropical forests. *Ecol. Res.* 24 1179–1186. 10.1007/s11284-009-0599-0

[B68] Pond S. L. K. Muse S. V. (2005). “HyPhy: hypothesis testing using phylogenies,” in *Statistical Methods in Molecular Evolution*, ed. Nielsen R. (New York, NY: Springer New York), 125–181. 10.1007/0-387-27733-1_6

[B69] Rambaut A. (2012). *FigTree v1. 4.* Available at: http://tree.bio.ed.ac.uk/software/figtree/ (accessed 28 April 2014).

[B70] Rieseberg L. H. Brouillet L. (1994). Are many plant species paraphyletic? *Taxon* 43 21–32. 10.2307/1223457

[B71] Rodriguez-Buritica S. Orjuela M. A. Galeano G. (2005). Demography and life history of *Geonoma orbignyana*: an understory palm used as foliage in Colombia. *For. Ecol. Manag.* 211 329–340. 10.1016/j.foreco.2005.02.052

[B72] Roncal J. (2006). Habitat differentiation of sympatric *Geonoma macrostachys* (Arecaceae) varieties in Peruvian lowland forests. *J. Trop. Ecol.* 22 483–486. 10.1017/s0266467406003270

[B73] Roncal J. Blach-Overgaard A. Borchsenius F. Balslev H. Svenning J. C. (2011). A dated phylogeny complements macroecological analysis to explain the diversity patterns in *Geonoma* (Arecaceae). *Biotropica* 43 324–334. 10.1111/j.1744-7429.2010.00696.x

[B74] Roncal J. Borchsenius F. Asmussen-Lange C. B. Balslev H. (2010). “Divergence times in the tribe Geonomateae (Arecaceae) coincide with tertiary geological events,” in *Diversity, Phylogeny and Evolution of the Monocotyledons*, eds Seberg O. Petersen G. Barfod A. S. Davis J. I. (Aarhus: Aarhus University Press), 245–265.

[B75] Roncal J. Francisco-Ortega J. Asmussen C. B. Lewis C. E. (2005). Molecular phylogenetics of tribe Geonomeae (Arecaceae) using nuclear DNA sequences of Phosphoribulokinase and RNA Polymerase II. *Syst. Bot.* 30 275–283. 10.1600/0363644054223620

[B76] Roncal J. Francisco-Ortega J. Lewis C. E. (2007). An evaluation of the taxonomic distinctness of two *Geonoma macrostachys* (Arecaceae) varieties based on intersimple sequence repeat (ISSR) variation. *Bot. J. Linn. Soc.* 153 381–392. 10.1111/j.1095-8339.2007.00619.x

[B77] Roncal J. Henderson A. Borchsenius F. Cardoso S. R. S. Balslev H. (2012). Can phylogenetic signal, character displacement, or random phenotypic drift explain the morphological variation in the genus *Geonoma* (Arecaceae)? *Biol. J. Linn. Soc.* 106 528–539. 10.1111/j.1095-8312.2012.01879.x

[B78] Roncal J. Zona S. Lewis C. E. (2008). Molecular phylogenetic studies of Caribbean palms (Arecaceae) and their relationships to biogeography and conservation. *Bot. Rev.* 74 78–102. 10.1007/s12229-008-9005-9

[B79] Sampaio M. B. Scariot A. (2008). Growth and reproduction of the understory palm *Geonoma schottiana* Mart. in the gallery forest in Central Brazil. *Rev. Bras. Bot.* 31 433–442. 10.1590/S0100-84042008000300007

[B80] Sanín M. J. Kissling W. D. Bacon C. D. Borchsenius F. Galeano G. Svenning J.-C. (2016). The neogene rise of the tropical Andes facilitated diversification of wax palms (*Ceroxylon*: Arecaceae) through geographical colonization and climatic niche separation. *Bot. J. Linn. Soc.* 182 303–317. 10.1111/boj.12419

[B81] Sass C. Iles W. J. D. Barrett C. F. Smith S. Y. Specht C. D. (2016). Revisiting the Zingiberales: using multiplexed exon capture to resolve ancient and recent phylogenetic splits in a charismatic plant lineage. *PeerJ* 4:e1584. 10.7717/peerj.1584 26819846 PMC4727956

[B82] Sayyari E. Mirarab S. (2016). Fast coalescent-based computation of local branch support from quartet frequencies. *Mol. Biol. Evol.* 33 1654–1668. 10.1093/molbev/msw079 27189547 PMC4915361

[B83] Singh R. Ong-Abdullah M. Low E.-T. L. Manaf M. A. A. Rosli R. Nookiah R. (2013). Oil palm genome sequence reveals divergence of interfertile species in Old and New worlds. *Nature* 500 335–339. 10.1038/nature12309 23883927 PMC3929164

[B84] Smeds L. Künstner A. (2011). ConDeTri-a content dependent read trimmer for Illumina data. *PLoS One* 6:e26314. 10.1371/journal.pone.0026314 22039460 PMC3198461

[B85] Stamatakis A. (2014). RAxML version 8: a tool for phylogenetic analysis and post-analysis of large phylogenies. *Bioinformatics* 30 1312–1313. 10.1093/bioinformatics/btu033 24451623 PMC3998144

[B86] Stauffer F. W. (1997). Estudio morfologico y taxonomico de *Geonoma spinescens* H. Wendl. ex Burret (Arecaceae) y descripcion de una nueva variedad. *Acta Bot. Venezuelica* 20 1–10.

[B87] Stauffer F. W. Asmussen C. B. Henderson A. Endress P. K. (2003). A revision of *Asterogyne* (Arecaceae: Arecoideae: Geonomeae). *Brittonia* 55 326–356. 10.1663/0007-196x(2003)055

[B88] Townsend J. P. (2007). Profiling phylogenetic informativeness. *Syst. Biol.* 56 222–231. 10.1080/10635150701311362 17464879

[B89] Uhl N. W. Dransfield J. (1987). *Genera Palmarum. A Classification of Palms Based on the Work of Harold E. Moore, Jr.* Lawrence, KS: Allen Press.

[B90] Uthaipaisanwong P. Chanprasert J. Shearman J. R. Sangsrakru D. Yoocha T. Jomchai N. (2012). Characterization of the chloroplast genome sequence of oil palm (*Elaeis guineensis* Jacq.). *Gene* 500 172–180. 10.1016/j.gene.2012.03.061 22487870

[B91] Vormisto J. Svenning J. Hall P. Balslev H. (2004). Diversity and dominance in palm (Arecaceae) communities in terra firme forests in the western Amazon basin. *J. Ecol.* 92 577–588. 10.1111/j.0022-0477.2004.00904.x

[B92] Wessels Boer J. G. (1968). The geonomoid palms. *Meded. Van Het Bot. Mus. En Herb. Van Rijksuniv. Te Utrecht* 282 1–202. 10.3732/ajb.89.11.1772 21665604

[B93] Whitney K. D. Ahern J. R. Campbell L. G. Albert L. P. King M. S. (2010). Patterns of hybridization in plants. *Perspect. Plant Ecol. Evol. Syst.* 12 175–182.

[B94] Wilson M. A. Gaut B. Clegg M. T. (1990). Chloroplast DNA evolves slowly in the palm family (Arecaceae). *Mol. Biol. Evol.* 7 303–314. 1974691 10.1093/oxfordjournals.molbev.a040605

[B95] Wu C. (2001). The genic view of the process of speciation. *J. Evol. Biol.* 14 851–865. 18579476

[B96] Yang M. Zhang X. Liu G. Yin Y. Chen K. Yun Q. (2010). The complete chloroplast genome sequence of date palm (*Phoenix dactylifera* L.). *PLoS One* 5:e12762. 10.1371/journal.pone.0012762 20856810 PMC2939885

[B97] Zona S. (1995). A revision of *Calyptronoma*. *Principes* 39 140–151.

