# Supplementary material for: Targeted Capture of Hundreds of Nuclear Genes Unravels Phylogenetic Relationships of the Diverse Neotropical Palm Tribe Geonomateae
Source: Front Plant Sci. 2019 Jul 12;10:864. doi: 10.3389/fpls.2019.00864 (PMC6640726; doi:10.3389/fpls.2019.00864)
Supplement: Supplementary file 3 [file Data_Sheet_1.docx]

**Supplementary Material and Method**

**Estimation of phylogenetic informativeness using TAPIR**

Estimation of phylogenetic informativeness was performed on the following selection of species:

| Species | Sample |
| --- | --- |
| *Asterogyne guianensis* | 2493 |
| *Asterogyne martiana* | IO503 |
| *Bactris gasipoes* | 743 |
| *Ceroxylon alpinum* | 523B |
| *Calyptrogyne anomala* | AC07 |
| *Calyptrogyne osensis* | 2564 |
| *Cocos nucifera* | 984 |
| *Geonoma deversa* | 1823 |
| *Geonoma deversa* | CB10 |
| *Geonoma deversa* | OL082 |
| *Geonoma deversa* | OL101_A |
| *Geonoma ferruginea* | 2534 |
| *Geonoma stricta* var *trailii* | AC60 |
| *Licuala distans* | 1017 |
| *Licuala merguensis* | 1011 |
| *Pholidostachys dactyloides* | 1119 |
| *Pholidostachys synanthera* subsp *robusta* | 1804 |
| *Socratea exorrhiza* | 3125 |
| *Welfia regia* | 3007 |
| *Wettinia maynensis* | 296B |

Gene trees were inferred for the 3,297 genomic regions which had no missing data i.e. had DNA sequence data available for all of the 20 selected samples. Gene tree estimation was performed in RaxML 8.2.10 (Stamatakis, 2014), using the GTR+GAMMA model of substitution. Bootstrapping was run with the -autoMRE option, which automatically stops the 1,000 bootstrap replicate searches if the support values converge earlier. We then computed the mean bootstrap value of each gene tree. All genes with a mean boostrap per gene tree >60 were concatenated into a single alignment (total length of 1,546,401 bp) which was then used to infer a phylogenetic tree in RaxML under the GTR+GAMMA model of substitution and with the -autoMRE option to perform bootstrap replicate searches. The maximum likelihood tree was subsequently time-calibrated using Penalized Likelihood in the program TreePL (Smith and O’meara, 2012). We chose this method because it is considerably faster and the purpose was to be able to compute phylogenetic informativeness over broad time slices rather than a obtaining a very precise estimation of node ages. Divergence time estimation was performed using the following calibration points:

1) The fossil *Sabal carolinensis* for the root node, following (Couvreur et al., 2011)

Minimum age = 85.8 My; Maximum age = 88.8 My

2) Divergence between Cocos and Bactris, following Couvreur et al. (2011).

Minimum age = 54.8 My; Maximum age = 60.79 My

3) Secondary calibration for crown Geonomateae using the estimate from (Roncal et al., 2010)⁠

Minimum age = 21.2 My; Maximum age = 33.5 My

4) Secondary calibration for crown *Geonoma* using the estimate from Roncal et al. (2010).

Minimum age = 11.9 My; Maximum age = 19.5 My

The resulting dated phylogeny in My is shown below (node numbers indicate age estimates in My):


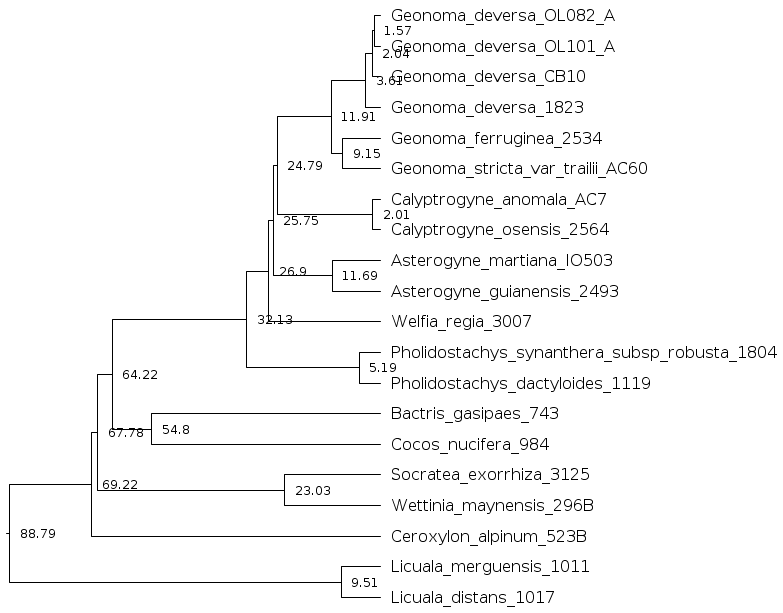


This dated phylogenetic tree was used to estimate the phylogenetic informativeness of the 3,287 genes over 5 time intervals (1-5 My, 5-15 My, 15-25 My, 25-50 My, 50-89 My) in TAPIR (Faircloth et al., 2012).

References

Couvreur, T. L. P., Forest, F., and Baker, W. J. (2011). Origin and global diversification patterns of tropical rain forests: inferences from a complete genus-level phylogeny of palms. *BMC biology* 9, 44. doi:10.1186/1741-7007-9-44.

Faircloth, B. C., Chang, J., and Alfaro, M. E. (2012). TAPIR enables high-throughput estimation and comparison of phylogenetic informativeness using locus-specific substitution models. *arXiv preprint arXiv:1202.1215*.

Roncal, J., Borchsenius, F., Asmussen-Lange, C. B., and Balslev, H. (2010). “Divergence Times in the Tribe Geonomateae (Arecaceae) coincide with Tertiary Geological Events,” in *Diversity, Phylogeny and Evolution in the Monocoltiledons*, 245–265.

Smith, S. A., and O’meara, B. C. (2012). treePL: divergence time estimation using penalized likelihood for large phylogenies. *Bioinformatics* 28, 2689–2690.

Stamatakis, A. (2014). RAxML version 8: a tool for phylogenetic analysis and post-analysis of large phylogenies. *Bioinformatics* 30, 1312–1313.
